# Diaqua­bis[4-(dimethyl­amino)benzoato-κ*O*](isonicotinamide-κ*N* ^1^)manganese(II)

**DOI:** 10.1107/S1600536809027093

**Published:** 2009-08-08

**Authors:** Tuncer Hökelek, Hakan Dal, Barış Tercan, Özgür Aybirdi, Hacali Necefoğlu

**Affiliations:** a Department of Physics, Hacettepe University, 06800 Beytepe, Ankara, Turkey; b Department of Chemistry, Faculty of Science, Anadolu University, 26470 Yenibağlar, Eskişehir, Turkey; c Department of Physics, Karabük University, 78050, Karabük, Turkey; d Department of Chemistry, Kafkas University, 63100 Kars, Turkey

## Abstract

The title Mn^II^ complex, [Mn(C_9_H_10_NO_2_)_2_(C_6_H_6_N_2_O)(H_2_O)_2_], contains two 4-(dimethylamino)benzoate (DMAB) anions, one isonicotinamide (INA) ligand and two coordinated water mol­ecules. One of the DMAB anions acts as a bidentate ligand, while the other is monodentate. The four O atoms in the equatorial plane around the Mn atom form a highly distorted square-planar arrangement, while the distorted octa­hedral coordination geometry is completed by the N atom of the INA ligand and the O atom of the second water mol­ecule in the axial positions. In the crystal structure, strong inter­molecular O—H⋯O, O—H⋯N and N—H⋯O hydrogen bonds link the mol­ecules into a two-dimensional network parallel to (010). Two weak C—H⋯π inter­actions are also found.

## Related literature

For general backgroud, see: Adiwidjaja *et al.* (1978 ▶); Amiraslanov *et al.* (1979 ▶); Antolini *et al.* (1982 ▶); Antsyshkina *et al.* (1980 ▶); Bigoli *et al.* (1972 ▶); Catterick *et al.* (1974 ▶); Chen & Chen (2002 ▶); Hauptmann *et al.* (2000 ▶); Krishnamachari (1974 ▶); Shnulin *et al.* (1981 ▶). For related structures, see: Hökelek *et al.* (2009*a* ▶,*b* ▶,*c* ▶,*d* ▶,*e* ▶,*f* ▶,*g* ▶); Özbek *et al.* (2009 ▶); Tercan *et al.* (2009 ▶).
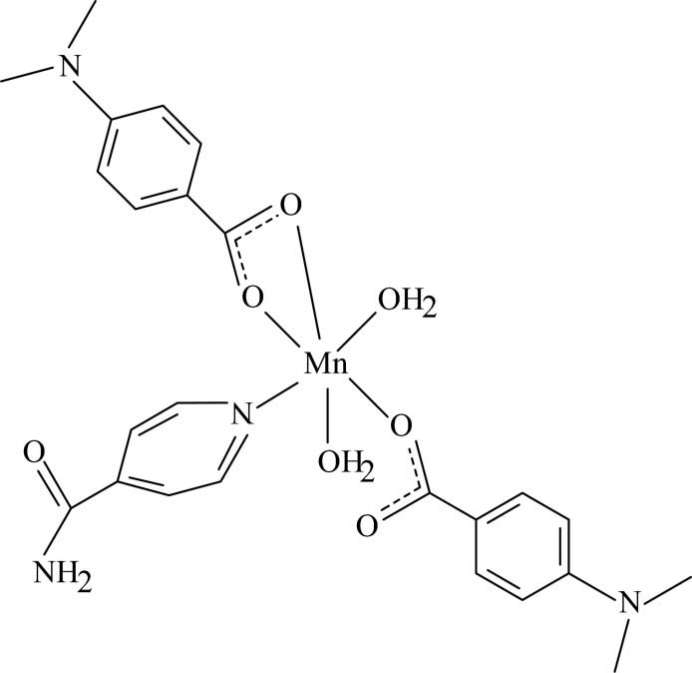


## Experimental

### 

#### Crystal data

[Mn(C_9_H_10_NO_2_)_2_(C_6_H_6_N_2_O)(H_2_O)_2_]
*M* *_r_* = 541.46
Monoclinic, 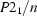

*a* = 6.9120 (2) Å
*b* = 45.1365 (5) Å
*c* = 8.1506 (2) Å
β = 93.889 (1)°
*V* = 2537.0 (1) Å^3^
*Z* = 4
Mo *K*α radiation
μ = 0.57 mm^−1^
*T* = 100 K
0.34 × 0.30 × 0.26 mm

#### Data collection

Bruker Kappa APEXII CCD area-detector diffractometer
Absorption correction: multi-scan (*SADABS*; Bruker, 2005 ▶) *T*_min_ = 0.822, *T*_max_ = 0.860
22933 measured reflections
6311 independent reflections
5980 reflections with *I* > 2σ(*I*)
*R*_int_ = 0.025

#### Refinement

*R*[*F* ^2^ > 2σ(*F* ^2^)] = 0.074
*wR*(*F* ^2^) = 0.147
*S* = 1.38
6311 reflections
344 parameters
6 restraints
H atoms treated by a mixture of independent and constrained refinement
Δρ_max_ = 0.50 e Å^−3^
Δρ_min_ = −1.15 e Å^−3^

### 

Data collection: *APEX2* (Bruker, 2007 ▶); cell refinement: *SAINT* (Bruker, 2007 ▶); data reduction: *SAINT*; program(s) used to solve structure: *SHELXS97* (Sheldrick, 2008 ▶); program(s) used to refine structure: *SHELXL97* (Sheldrick, 2008 ▶); molecular graphics: *ORTEP-3 for Windows* (Farrugia, 1997 ▶); software used to prepare material for publication: *WinGX* (Farrugia, 1999 ▶).

## Supplementary Material

Crystal structure: contains datablocks I, global. DOI: 10.1107/S1600536809027093/dn2464sup1.cif

Structure factors: contains datablocks I. DOI: 10.1107/S1600536809027093/dn2464Isup2.hkl

Additional supplementary materials:  crystallographic information; 3D view; checkCIF report

## Figures and Tables

**Table 1:** Hydrogen-bond geometry (Å, °)

*D*—H⋯*A*	*D*—H	H⋯*A*	*D*⋯*A*	*D*—H⋯*A*
N2—H2*A*⋯O4^i^	0.86	2.18	3.025 (4)	167
N2—H2*B*⋯O3^ii^	0.86	2.00	2.821 (4)	159
O6—H61⋯O2	0.92 (4)	1.78 (4)	2.672 (4)	162 (4)
O6—H62⋯O5^iii^	0.89 (4)	1.85 (4)	2.739 (4)	175 (4)
O7—H71⋯N3^iv^	0.91 (2)	1.96 (2)	2.838 (2)	161 (2)
O7—H72⋯O2^v^	0.88 (2)	1.80 (2)	2.671 (2)	170 (2)
C23—H23*B*⋯*Cg*2^vi^	0.96	2.90	3.649 (4)	136
C24—H24*A*⋯*Cg*3^vii^	0.96	2.74	3.594 (3)	149

Symmetry codes: (i) 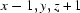; (ii) 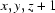; (iii) 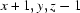; (iv) 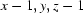; (v) 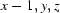; (vi) 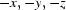; (vii) 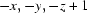. *Cg*2 and *Cg*3 are centroids of the C9–C14 and N1/C15–C19 rings, respectively.

**Table 2:** Comparison of the carboxylate bonds (Å) in the title compound with the corresponding values in related compounds

Compound	C1—O1	C1—O2	C8—O3	C8—O4
I^*a*^	1.261 (4)	1.267 (4)	1.277 (4)	1.270 (4)
II^*b*^	1.244 (4)	1.270 (4)		
III^*c*^	1.284 (2)	1.248 (2)		
	1.278 (2)	1.241 (2)		
IV^*d*^	1.267 (3)	1.258 (3)		
V^*e*^	1.263 (2)	1.240 (2)		
VI^*f*^	1.2611 (17)	1.2396 (19)		
VII^*g*^	1.2616 (17)	1.2435 (18)		
VIII^*h*^	1.2746 (18)	1.2675 (17)		
I*X*^*i*^	1.2682 (17)	1.2628 (17)	1.2743 (18)	1.2716 (18)
*X*^*j*^	1.265 (3)	1.265 (3)	1.278 (3)	1.271 (3)

Notes: (*a*) this work; (*b*) [Co(NA)_2_(H_2_O)_4_](C_7_H_4_FO_2_)_2_ (Özbek *et al.*, 2009 ▶); (*c*) [Zn(NA)_2_C_8_H_8_NO_2_)_2_] (Tercan *et al.*, 2009 ▶); (*d*) [Ni(NA)_2_(C_7_H_4_ClO_2_)_2_(H_2_O)_2_] (Hökelek *et al.*, 2009*a* ▶); (*e*) [Zn(DENA)_2_(C_7_H_4_BrO_2_)_2_(H_2_O)_2_] (Hökelek *et al.*, 2009*b* ▶); (*f*) [Mn(DENA)_2_(C_7_H_4_BrO_2_)_2_(H_2_O)_2_] (Hökelek *et al.*, 2009*c* ▶); (*g*) [Ni(DENA)_2_(C_7_H_4_(ClO_2_)_2_(H_2_O)_2_] (Hökelek *et al.*, 2009*d* ▶); (*h*) [Ni(NA)_2_(C_8_H_8_NO_2_)_2_(H_2_O)_2_] (Hökelek *et al.*, 2009*e* ▶); (*i*) [Co(NA)_2_(C_9_H_10_NO_2_)_2_(H_2_O)_2_] (Hökelek *et al.*, 2009*f* ▶); (*j*) [Zn(NA)_2_((C_9_H_10_NO_2_)_2_(H_2_O)_2_] (Hökelek *et al.*, 2009*g* ▶).

**Fig. 1.**
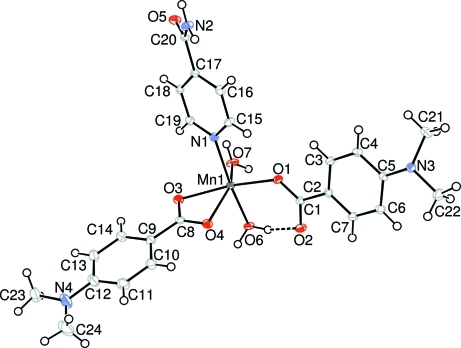
The molecular structure of the title molecule with the atom-numbering scheme. Displacement ellipsoids are drawn at the 50% probability level. Hydrogen bond is shown as dashed line.

## supplementary crystallographic information

### Comment

Nicotinamide (NA) is one form of niacin. A deficiency of this vitamin leads to loss of copper from the body, known as pellagra disease. Victims of pellagra show unusually high serum and urinary copper levels (Krishnamachari, 1974). The nicotinic acid derivative *N*,*N*-Diethylnicotinamide (DENA) is an important respiratory stimulant (Bigoli *et al.*, 1972). Transition metal complexes with biochemical molecules show interesting physical and/or chemical properties, through which they may find applications in biological systems (Antolini *et al.*, 1982). Some benzoic acid derivatives, such as 4-aminobenzoic acid, have been extensively reported in coordination chemistry, as bifunctional organic ligands, due to the varieties of their coordination modes (Chen & Chen, 2002; Amiraslanov *et al.*, 1979; Hauptmann *et al.*, 2000).

The structure-function-coordination relationships of the arylcarboxylate ion in Mn^II^ complexes of benzoic acid derivatives may also change depending on the nature and position of the substituted groups on the benzene ring, the nature of the additional ligand molecule or solvent, and the pH and temperature of synthesis, as in Zn^II^ complexes (Shnulin *et al.*, 1981; Antsyshkina *et al.*, 1980; Adiwidjaja *et al.*, 1978). When pyridine and its derivatives are used instead of water molecules, the structure is completely different (Catterick *et al.*, 1974).

The structure determination of the title compound, (I), a manganese complex with two 4-dimethylaminobenzoate (DMAB) and one isonicotinamide (INA) ligands and two water molecules, was undertaken in order to determine the properties of the ligands and also to compare the results obtained with those reported previously.

In the monomeric title complex, (I), the Mn atom is surrounded by two DMAB and INA ligands and two water molecules. One of the DMAB ions acts as a bidentate ligand, while the other and INA are monodentate ligands (Fig. 1). The four O atoms (O1, O3, O4 and O6 atoms) in the equatorial plane around the Mn atom form a highly distorted square-planar arrangement, while the distorted octahedral coordination is completed by the N atom of the INA ligand (N1) and the O atom of the water molecule (O6) in the axial positions (Fig. 1).

The near equality of the C1—O1 [1.261 (4) Å], C1—O2 [1.267 (4) Å], C8—O3 [1.277 (4) Å] and C8—O4 [1.270 (4) Å], bonds in the carboxylate group indicates a delocalized bonding arrangement, rather than localized single and double bonds, and may be compared with the corresponding distances (Table 1). In (I), the average Mn—O bond length is 2.196 (2) Å and the Mn atom is displaced out of the least-squares planes of the carboxylate groups (O1/C1/O2) and (O3/C8/O4) by 0.525 (1) Å and 0.020 (1) Å, respectively. The dihedral angle between the planar carboxylate groups and the adjacent benzene rings A (C2—C7) and B (C9—C14) are 5.97 (2)° and 2.32 (2)°, respectively, while those between rings A, B and C (N1/C15—C19) are A/B = 65.38 (3), A/C = 10.20 (3) and B/C = 73.72 (3) °. Intramolecular O—H···O hydrogen bond (Table 2) results in the formation of a six-membered ring D (Mn1/O1/O2/O6/C1/H61) adopting twisted conformation.

In the crystal structure, strong intermolecular O—H···O, O—H···N and N—H···O hydrogen bonds (Table 2) link the molecules into a two-dimensional network, in which they may be effective in the stabilization of the structure. Two weak C—H···π interactions (Table 2) are also found.

### Experimental

The title compound was prepared by the reaction of MnSO_4_.H_2_O (0.85 g, 5 mmol) in H_2_O (30 ml) and INA (1.22 g, 10 mmol) in H_2_O (20 ml) with sodium *p*-dimethylaminobenzoate (1.88 g, 10 mmol) in H_2_O (50 ml). The mixture was filtered and set aside to crystallize at ambient temperature for one week, giving colorless single crystals.

### Refinement

H atoms of water molecules were located in difference Fourier maps and refined isotropically, with restrains of O6—H61 = 0.92 (4), O6—H62 = 0.89 (4), O7—H71 = 0.909 (17), O7—H72 = 0.876 (19) Å and H61—O6—H62 = 107 (3) and H71—O7—H72 = 107 (3) °. The remaining H atoms were positioned geometrically with N—H = 0.86 Å (for NH_2_) and C—H = 0.93 and 0.96 Å, for aromatic and methyl H atoms, respectively, and constrained to ride on their parent atoms, with *U*_iso_(H) = *xU*_eq_(C,*N*), where *x* = 1.5 for methyl H and *x* = 1.2 for all other H atoms.

### Crystal data

**Table:**

[Mn(C_9_H_10_NO_2_)_2_(C_6_H_6_N_2_O)(H_2_O)_2_]	*F*(000) = 1132
*M**_r_* = 541.46	*D*_x_ = 1.418 Mg m^−^^3^
Monoclinic, *P*2_1_/*n*	Mo *K*α radiation, λ = 0.71073 Å
Hall symbol: -P 2yn	Cell parameters from 9856 reflections
*a* = 6.9120 (2) Å	θ = 2.6–28.4°
*b* = 45.1365 (5) Å	µ = 0.57 mm^−^^1^
*c* = 8.1506 (2) Å	*T* = 100 K
β = 93.889 (1)°	Block, colorless
*V* = 2537.0 (1) Å^3^	0.34 × 0.30 × 0.26 mm
*Z* = 4	

### Data collection

**Table:**

Bruker Kappa APEXII CCD area-detector diffractometer	6311 independent reflections
Radiation source: fine-focus sealed tube	5980 reflections with *I* > 2σ(*I*)
graphite	*R*_int_ = 0.025
φ and ω scans	θ_max_ = 28.5°, θ_min_ = 1.8°
Absorption correction: multi-scan (*SADABS*; Bruker, 2005)	*h* = −9→8
*T*_min_ = 0.822, *T*_max_ = 0.860	*k* = −60→59
22933 measured reflections	*l* = −10→10

### Refinement

**Table:**

Refinement on *F*^2^	Primary atom site location: structure-invariant direct methods
Least-squares matrix: full	Secondary atom site location: difference Fourier map
*R*[*F*^2^ > 2σ(*F*^2^)] = 0.074	Hydrogen site location: inferred from neighbouring sites
*wR*(*F*^2^) = 0.147	H atoms treated by a mixture of independent and constrained refinement
*S* = 1.38	*w* = 1/[σ^2^(*F*_o_^2^) + (0.*P*)^2^ + 9.0299*P*] where *P* = (*F*_o_^2^ + 2*F*_c_^2^)/3
6311 reflections	(Δ/σ)_max_ < 0.001
344 parameters	Δρ_max_ = 0.50 e Å^−^^3^
6 restraints	Δρ_min_ = −1.15 e Å^−^^3^

### Special details

**Table:**

Geometry. All e.s.d.'s (except the e.s.d. in the dihedral angle between two l.s. planes) are estimated using the full covariance matrix. The cell e.s.d.'s are taken into account individually in the estimation of e.s.d.'s in distances, angles and torsion angles; correlations between e.s.d.'s in cell parameters are only used when they are defined by crystal symmetry. An approximate (isotropic) treatment of cell e.s.d.'s is used for estimating e.s.d.'s involving l.s. planes.
Refinement. Refinement of *F*^2^ against ALL reflections. The weighted *R*-factor *wR* and goodness of fit *S* are based on *F*^2^, conventional *R*-factors *R* are based on *F*, with *F* set to zero for negative *F*^2^. The threshold expression of *F*^2^ > σ(*F*^2^) is used only for calculating *R*-factors(gt) *etc*. and is not relevant to the choice of reflections for refinement. *R*-factors based on *F*^2^ are statistically about twice as large as those based on *F*, and *R*- factors based on ALL data will be even larger.

### Fractional atomic coordinates and isotropic or equivalent isotropic displacement parameters (Å^2^)

**Table:**

	*x*	*y*	*z*	*U*_iso_*/*U*_eq_	
Mn1	0.89626 (8)	0.138233 (11)	0.52423 (6)	0.01186 (12)	
O1	1.0707 (4)	0.16630 (6)	0.6803 (3)	0.0163 (5)	
O2	1.3091 (4)	0.17803 (6)	0.5212 (3)	0.0168 (5)	
O3	0.7933 (3)	0.09672 (5)	0.4009 (3)	0.0154 (5)	
O4	1.0942 (4)	0.09799 (5)	0.5074 (3)	0.0145 (5)	
O5	0.1617 (4)	0.11645 (6)	1.0883 (3)	0.0179 (5)	
O6	1.0332 (4)	0.15667 (6)	0.3078 (3)	0.0154 (5)	
H61	1.137 (5)	0.1657 (10)	0.363 (5)	0.034 (14)*	
H62	1.081 (7)	0.1434 (9)	0.240 (5)	0.042 (15)*	
O7	0.6603 (4)	0.16490 (6)	0.4240 (3)	0.0184 (5)	
H71	0.694 (6)	0.1821 (6)	0.375 (5)	0.029 (13)*	
H72	0.541 (3)	0.1671 (9)	0.452 (5)	0.024*	
N1	0.7162 (4)	0.12666 (6)	0.7352 (3)	0.0132 (6)	
N2	0.4078 (5)	0.10213 (7)	1.2669 (4)	0.0176 (6)	
H2A	0.3322	0.0996	1.3453	0.021*	
H2B	0.5301	0.0988	1.2834	0.021*	
N3	1.6781 (4)	0.21772 (6)	1.2368 (4)	0.0145 (6)	
N4	1.0784 (6)	−0.03194 (8)	0.1892 (5)	0.0333 (9)	
C1	1.2368 (5)	0.17669 (7)	0.6597 (4)	0.0129 (6)	
C2	1.3526 (5)	0.18809 (7)	0.8083 (4)	0.0126 (6)	
C3	1.2728 (5)	0.18913 (8)	0.9603 (4)	0.0148 (6)	
H3	1.1459	0.1827	0.9687	0.018*	
C4	1.3773 (5)	0.19945 (8)	1.0992 (4)	0.0158 (7)	
H4	1.3192	0.2003	1.1987	0.019*	
C5	1.5709 (5)	0.20875 (7)	1.0916 (4)	0.0125 (6)	
C6	1.6523 (5)	0.20724 (8)	0.9390 (4)	0.0153 (7)	
H6	1.7804	0.2130	0.9307	0.018*	
C7	1.5446 (5)	0.19722 (8)	0.8003 (4)	0.0158 (7)	
H7	1.6014	0.1966	0.7001	0.019*	
C8	0.9572 (5)	0.08403 (7)	0.4297 (4)	0.0131 (6)	
C9	0.9864 (5)	0.05360 (8)	0.3708 (4)	0.0158 (7)	
C10	1.1647 (6)	0.03925 (8)	0.4017 (5)	0.0199 (7)	
H10	1.2647	0.0489	0.4623	0.024*	
C11	1.1946 (6)	0.01099 (9)	0.3434 (5)	0.0245 (8)	
H11	1.3143	0.0019	0.3657	0.029*	
C12	1.0473 (7)	−0.00428 (8)	0.2511 (5)	0.0243 (8)	
C13	0.8674 (6)	0.01007 (9)	0.2229 (5)	0.0248 (8)	
H13	0.7664	0.0004	0.1639	0.030*	
C14	0.8381 (6)	0.03848 (8)	0.2817 (5)	0.0203 (7)	
H14	0.7180	0.0475	0.2613	0.024*	
C15	0.7809 (5)	0.13009 (8)	0.8924 (4)	0.0161 (7)	
H15	0.9075	0.1366	0.9153	0.019*	
C16	0.6671 (5)	0.12440 (8)	1.0228 (4)	0.0157 (7)	
H16	0.7181	0.1264	1.1308	0.019*	
C17	0.4757 (5)	0.11571 (7)	0.9890 (4)	0.0129 (6)	
C18	0.4073 (5)	0.11246 (7)	0.8254 (4)	0.0130 (6)	
H18	0.2797	0.1068	0.7988	0.016*	
C19	0.5315 (5)	0.11775 (8)	0.7038 (4)	0.0149 (6)	
H19	0.4858	0.1150	0.5949	0.018*	
C20	0.3358 (5)	0.11123 (7)	1.1209 (4)	0.0126 (6)	
C21	1.5745 (6)	0.23707 (9)	1.3459 (5)	0.0230 (8)	
H21A	1.6569	0.2415	1.4424	0.034*	
H21B	1.4595	0.2272	1.3772	0.034*	
H21C	1.5395	0.2552	1.2895	0.034*	
C22	1.8760 (6)	0.22797 (9)	1.2178 (5)	0.0223 (8)	
H22A	1.9377	0.2324	1.3239	0.033*	
H22B	1.8725	0.2455	1.1506	0.033*	
H22C	1.9476	0.2127	1.1663	0.033*	
C23	0.9198 (8)	−0.04870 (10)	0.1085 (6)	0.0397 (12)	
H23A	0.9672	−0.0675	0.0732	0.060*	
H23B	0.8661	−0.0379	0.0148	0.060*	
H23C	0.8211	−0.0519	0.1841	0.060*	
C24	1.2663 (8)	−0.04584 (10)	0.2133 (7)	0.0429 (13)	
H24A	1.2638	−0.0647	0.1583	0.064*	
H24B	1.2976	−0.0487	0.3288	0.064*	
H24C	1.3625	−0.0334	0.1690	0.064*	

### Atomic displacement parameters (Å^2^)

**Table:**

	*U*^11^	*U*^22^	*U*^33^	*U*^12^	*U*^13^	*U*^23^
Mn1	0.0110 (2)	0.0148 (2)	0.0100 (2)	−0.0003 (2)	0.00228 (17)	−0.00047 (19)
O1	0.0136 (12)	0.0213 (12)	0.0145 (12)	−0.0047 (10)	0.0052 (9)	−0.0037 (9)
O2	0.0117 (12)	0.0261 (13)	0.0127 (12)	−0.0010 (10)	0.0017 (9)	−0.0015 (10)
O3	0.0111 (11)	0.0195 (12)	0.0155 (12)	0.0009 (9)	−0.0003 (9)	−0.0026 (9)
O4	0.0144 (12)	0.0184 (12)	0.0106 (11)	0.0001 (9)	0.0008 (9)	−0.0019 (9)
O5	0.0137 (12)	0.0263 (13)	0.0136 (12)	0.0016 (10)	0.0012 (9)	−0.0031 (10)
O6	0.0128 (12)	0.0203 (12)	0.0135 (12)	0.0005 (10)	0.0047 (9)	0.0006 (9)
O7	0.0131 (12)	0.0230 (13)	0.0197 (13)	0.0048 (10)	0.0065 (10)	0.0062 (10)
N1	0.0142 (14)	0.0126 (13)	0.0130 (13)	0.0003 (11)	0.0024 (11)	0.0011 (10)
N2	0.0147 (14)	0.0267 (16)	0.0117 (14)	−0.0015 (12)	0.0022 (11)	0.0012 (11)
N3	0.0144 (14)	0.0161 (14)	0.0132 (13)	−0.0027 (11)	0.0021 (11)	−0.0017 (11)
N4	0.051 (2)	0.0158 (16)	0.034 (2)	0.0016 (16)	0.0109 (18)	−0.0041 (14)
C1	0.0110 (15)	0.0138 (15)	0.0140 (15)	0.0012 (12)	0.0013 (12)	−0.0011 (12)
C2	0.0154 (16)	0.0112 (14)	0.0112 (15)	−0.0013 (12)	0.0007 (12)	0.0003 (11)
C3	0.0125 (16)	0.0163 (16)	0.0158 (16)	−0.0007 (13)	0.0038 (12)	0.0003 (12)
C4	0.0183 (17)	0.0173 (16)	0.0121 (16)	−0.0019 (13)	0.0040 (13)	−0.0010 (12)
C5	0.0144 (16)	0.0108 (14)	0.0122 (15)	0.0017 (12)	0.0005 (12)	−0.0005 (11)
C6	0.0152 (16)	0.0174 (16)	0.0134 (16)	−0.0010 (13)	0.0021 (13)	−0.0003 (12)
C7	0.0143 (16)	0.0191 (16)	0.0145 (16)	0.0007 (13)	0.0043 (13)	−0.0004 (12)
C8	0.0148 (16)	0.0142 (15)	0.0108 (15)	0.0007 (12)	0.0044 (12)	0.0021 (12)
C9	0.0189 (17)	0.0140 (15)	0.0150 (16)	−0.0003 (13)	0.0036 (13)	0.0007 (12)
C10	0.0214 (19)	0.0175 (17)	0.0210 (18)	0.0015 (14)	0.0036 (14)	0.0010 (14)
C11	0.026 (2)	0.0191 (18)	0.029 (2)	0.0068 (15)	0.0057 (16)	0.0030 (15)
C12	0.039 (2)	0.0161 (17)	0.0192 (18)	0.0007 (16)	0.0100 (16)	0.0006 (14)
C13	0.032 (2)	0.0189 (18)	0.024 (2)	−0.0049 (16)	0.0060 (16)	−0.0034 (15)
C14	0.0223 (19)	0.0186 (17)	0.0205 (18)	0.0003 (14)	0.0046 (14)	−0.0028 (14)
C15	0.0104 (15)	0.0208 (17)	0.0170 (16)	−0.0004 (13)	0.0005 (12)	0.0011 (13)
C16	0.0171 (17)	0.0181 (16)	0.0115 (15)	−0.0005 (13)	−0.0018 (12)	−0.0011 (12)
C17	0.0166 (16)	0.0108 (14)	0.0118 (15)	0.0012 (12)	0.0046 (12)	0.0001 (11)
C18	0.0117 (15)	0.0137 (15)	0.0136 (15)	−0.0022 (12)	−0.0002 (12)	−0.0010 (12)
C19	0.0166 (17)	0.0165 (16)	0.0113 (15)	0.0000 (13)	0.0002 (12)	−0.0012 (12)
C20	0.0140 (16)	0.0149 (15)	0.0091 (14)	−0.0016 (12)	0.0020 (12)	−0.0025 (11)
C21	0.025 (2)	0.0225 (19)	0.0216 (19)	−0.0009 (15)	0.0046 (15)	−0.0104 (14)
C22	0.0181 (18)	0.030 (2)	0.0183 (18)	−0.0063 (15)	0.0007 (14)	−0.0033 (15)
C23	0.059 (3)	0.018 (2)	0.044 (3)	−0.010 (2)	0.020 (2)	−0.0083 (18)
C24	0.062 (4)	0.020 (2)	0.048 (3)	0.012 (2)	0.018 (3)	−0.0008 (19)

### Geometric parameters (Å, °)

**Table:**

Mn1—O1	2.114 (2)	C7—C2	1.395 (5)
Mn1—O3	2.221 (2)	C7—H7	0.9300
Mn1—O4	2.284 (2)	C8—C9	1.473 (5)
Mn1—O6	2.219 (2)	C9—C10	1.400 (5)
Mn1—O7	2.144 (3)	C10—H10	0.9300
Mn1—N1	2.252 (3)	C11—C10	1.381 (5)
Mn1—C8	2.608 (3)	C11—C12	1.405 (6)
O1—C1	1.261 (4)	C11—H11	0.9300
O2—C1	1.267 (4)	C13—C12	1.408 (6)
O3—C8	1.277 (4)	C13—H13	0.9300
O4—C8	1.270 (4)	C14—C9	1.394 (5)
O5—C20	1.238 (4)	C14—C13	1.388 (5)
O6—H61	0.92 (4)	C14—H14	0.9300
O6—H62	0.89 (4)	C15—C16	1.388 (5)
O7—H71	0.909 (17)	C15—H15	0.9300
O7—H72	0.876 (19)	C16—H16	0.9300
N1—C15	1.337 (4)	C17—C16	1.389 (5)
N2—C20	1.324 (4)	C17—C18	1.392 (5)
N2—H2A	0.8600	C17—C20	1.507 (5)
N2—H2B	0.8600	C18—H18	0.9300
N3—C5	1.412 (4)	C19—N1	1.346 (4)
N3—C21	1.467 (5)	C19—C18	1.376 (5)
N3—C22	1.462 (5)	C19—H19	0.9300
N4—C12	1.369 (5)	C21—H21A	0.9600
N4—C23	1.453 (6)	C21—H21B	0.9600
N4—C24	1.444 (7)	C21—H21C	0.9600
C1—C2	1.497 (5)	C22—H22A	0.9600
C3—C2	1.391 (5)	C22—H22B	0.9600
C3—C4	1.382 (5)	C22—H22C	0.9600
C3—H3	0.9300	C23—H23A	0.9600
C4—H4	0.9300	C23—H23B	0.9600
C5—C4	1.407 (5)	C23—H23C	0.9600
C5—C6	1.401 (5)	C24—H24A	0.9600
C6—C7	1.387 (5)	C24—H24B	0.9600
C6—H6	0.9300	C24—H24C	0.9600
			
O1—Mn1—O3	158.49 (10)	O4—C8—O3	119.4 (3)
O1—Mn1—O4	101.13 (9)	O4—C8—C9	120.7 (3)
O1—Mn1—O6	89.82 (10)	O4—C8—Mn1	61.14 (17)
O1—Mn1—O7	106.33 (11)	C9—C8—Mn1	177.8 (3)
O1—Mn1—N1	90.10 (10)	C10—C9—C8	120.8 (3)
O1—Mn1—C8	130.01 (11)	C14—C9—C8	120.9 (3)
O3—Mn1—O4	58.45 (9)	C14—C9—C10	118.3 (3)
O3—Mn1—N1	88.47 (10)	C9—C10—H10	119.5
O3—Mn1—C8	29.30 (10)	C11—C10—C9	121.1 (4)
O4—Mn1—C8	29.15 (10)	C11—C10—H10	119.5
O6—Mn1—O3	95.59 (9)	C10—C11—C12	121.2 (4)
O6—Mn1—O4	87.77 (9)	C10—C11—H11	119.4
O6—Mn1—N1	168.98 (10)	C12—C11—H11	119.4
O6—Mn1—C8	91.77 (10)	N4—C12—C11	121.2 (4)
O7—Mn1—O3	95.10 (10)	N4—C12—C13	121.2 (4)
O7—Mn1—O4	150.12 (10)	C11—C12—C13	117.5 (3)
O7—Mn1—O6	80.84 (9)	C12—C13—H13	119.5
O7—Mn1—N1	88.61 (10)	C14—C13—C12	121.1 (4)
O7—Mn1—C8	123.23 (11)	C14—C13—H13	119.5
N1—Mn1—O4	103.05 (9)	C9—C14—H14	119.6
N1—Mn1—C8	96.71 (10)	C13—C14—C9	120.9 (4)
C1—O1—Mn1	129.0 (2)	C13—C14—H14	119.6
C8—O3—Mn1	92.4 (2)	N1—C15—C16	122.8 (3)
C8—O4—Mn1	89.7 (2)	N1—C15—H15	118.6
Mn1—O6—H61	98 (3)	C16—C15—H15	118.6
Mn1—O6—H62	116 (3)	C15—C16—C17	118.8 (3)
H61—O6—H62	107 (3)	C15—C16—H16	120.6
Mn1—O7—H71	116 (3)	C17—C16—H16	120.6
Mn1—O7—H72	132 (3)	C16—C17—C18	118.4 (3)
H71—O7—H72	107 (3)	C16—C17—C20	123.0 (3)
C15—N1—Mn1	122.6 (2)	C18—C17—C20	118.4 (3)
C15—N1—C19	117.9 (3)	C17—C18—H18	120.5
C19—N1—Mn1	119.4 (2)	C19—C18—C17	118.9 (3)
C20—N2—H2A	120.0	C19—C18—H18	120.5
C20—N2—H2B	120.0	N1—C19—C18	123.0 (3)
H2A—N2—H2B	120.0	N1—C19—H19	118.5
C5—N3—C21	115.4 (3)	C18—C19—H19	118.5
C5—N3—C22	116.3 (3)	O5—C20—N2	123.6 (3)
C22—N3—C21	112.0 (3)	O5—C20—C17	118.9 (3)
C12—N4—C23	120.6 (4)	N2—C20—C17	117.6 (3)
C12—N4—C24	120.6 (4)	N3—C21—H21A	109.5
C24—N4—C23	118.7 (4)	N3—C21—H21B	109.5
O1—C1—O2	123.6 (3)	N3—C21—H21C	109.5
O1—C1—C2	117.5 (3)	H21A—C21—H21B	109.5
O2—C1—C2	118.9 (3)	H21A—C21—H21C	109.5
C3—C2—C1	120.6 (3)	H21B—C21—H21C	109.5
C3—C2—C7	117.9 (3)	N3—C22—H22A	109.5
C7—C2—C1	121.5 (3)	N3—C22—H22B	109.5
C2—C3—H3	119.2	N3—C22—H22C	109.5
C4—C3—C2	121.6 (3)	H22A—C22—H22B	109.5
C4—C3—H3	119.2	H22A—C22—H22C	109.5
C3—C4—C5	120.7 (3)	H22B—C22—H22C	109.5
C3—C4—H4	119.7	N4—C23—H23A	109.5
C5—C4—H4	119.7	N4—C23—H23B	109.5
C4—C5—N3	119.7 (3)	N4—C23—H23C	109.5
C6—C5—C4	117.7 (3)	H23A—C23—H23B	109.5
C6—C5—N3	122.6 (3)	H23A—C23—H23C	109.5
C5—C6—H6	119.5	H23B—C23—H23C	109.5
C7—C6—C5	120.9 (3)	N4—C24—H24A	109.5
C7—C6—H6	119.5	N4—C24—H24B	109.5
C2—C7—H7	119.4	N4—C24—H24C	109.5
C6—C7—C2	121.2 (3)	H24A—C24—H24B	109.5
C6—C7—H7	119.4	H24A—C24—H24C	109.5
O3—C8—C9	119.8 (3)	H24B—C24—H24C	109.5
O3—C8—Mn1	58.31 (17)		
			
O3—Mn1—O1—C1	76.6 (4)	C21—N3—C5—C6	139.4 (3)
O4—Mn1—O1—C1	59.4 (3)	C22—N3—C5—C4	−178.1 (3)
O6—Mn1—O1—C1	−28.3 (3)	C22—N3—C5—C6	5.3 (5)
O7—Mn1—O1—C1	−108.7 (3)	C23—N4—C12—C11	173.7 (4)
N1—Mn1—O1—C1	162.7 (3)	C23—N4—C12—C13	−7.1 (6)
C8—Mn1—O1—C1	63.9 (3)	C24—N4—C12—C11	−1.9 (6)
O1—Mn1—O3—C8	−20.3 (4)	C24—N4—C12—C13	177.3 (4)
O4—Mn1—O3—C8	−0.29 (18)	O1—C1—C2—C3	−4.8 (5)
O6—Mn1—O3—C8	83.6 (2)	O1—C1—C2—C7	173.5 (3)
O7—Mn1—O3—C8	164.89 (19)	O2—C1—C2—C3	175.0 (3)
N1—Mn1—O3—C8	−106.6 (2)	O2—C1—C2—C7	−6.7 (5)
O1—Mn1—O4—C8	172.96 (19)	C4—C3—C2—C1	179.7 (3)
O3—Mn1—O4—C8	0.29 (18)	C4—C3—C2—C7	1.4 (5)
O6—Mn1—O4—C8	−97.66 (19)	C2—C3—C4—C5	−1.3 (5)
O7—Mn1—O4—C8	−30.5 (3)	N3—C5—C4—C3	−176.6 (3)
N1—Mn1—O4—C8	80.2 (2)	C6—C5—C4—C3	0.2 (5)
O1—Mn1—N1—C15	−20.9 (3)	N3—C5—C6—C7	177.4 (3)
O1—Mn1—N1—C19	154.6 (3)	C4—C5—C6—C7	0.7 (5)
O3—Mn1—N1—C15	137.6 (3)	C5—C6—C7—C2	−0.6 (5)
O3—Mn1—N1—C19	−46.9 (3)	C6—C7—C2—C1	−178.8 (3)
O4—Mn1—N1—C15	80.5 (3)	C6—C7—C2—C3	−0.5 (5)
O4—Mn1—N1—C19	−103.9 (3)	O3—C8—C9—C10	−179.8 (3)
O6—Mn1—N1—C15	−110.5 (5)	O3—C8—C9—C14	0.9 (5)
O6—Mn1—N1—C19	65.1 (6)	O4—C8—C9—C10	1.1 (5)
O7—Mn1—N1—C15	−127.3 (3)	O4—C8—C9—C14	−178.3 (3)
O7—Mn1—N1—C19	48.3 (3)	C8—C9—C10—C11	−178.4 (3)
C8—Mn1—N1—C15	109.4 (3)	C14—C9—C10—C11	0.9 (5)
C8—Mn1—N1—C19	−75.0 (3)	C12—C11—C10—C9	0.1 (6)
O1—Mn1—C8—O3	170.45 (17)	C10—C11—C12—N4	178.1 (4)
O1—Mn1—C8—O4	−9.0 (2)	C10—C11—C12—C13	−1.1 (6)
O3—Mn1—C8—O4	−179.5 (3)	C14—C13—C12—N4	−178.1 (4)
O4—Mn1—C8—O3	179.5 (3)	C14—C13—C12—C11	1.2 (6)
O6—Mn1—C8—O3	−98.29 (19)	C9—C14—C13—C12	−0.1 (6)
O6—Mn1—C8—O4	82.22 (19)	C13—C14—C9—C8	178.4 (3)
O7—Mn1—C8—O3	−18.1 (2)	C13—C14—C9—C10	−0.9 (5)
O7—Mn1—C8—O4	162.42 (17)	N1—C15—C16—C17	−2.2 (5)
N1—Mn1—C8—O3	74.7 (2)	C18—C17—C16—C15	1.3 (5)
N1—Mn1—C8—O4	−104.83 (19)	C20—C17—C16—C15	−175.3 (3)
Mn1—O1—C1—O2	18.6 (5)	C16—C17—C18—C19	0.5 (5)
Mn1—O1—C1—C2	−161.6 (2)	C20—C17—C18—C19	177.2 (3)
Mn1—O3—C8—O4	0.5 (3)	C16—C17—C20—O5	148.3 (3)
Mn1—O3—C8—C9	−178.7 (3)	C16—C17—C20—N2	−31.0 (5)
Mn1—O4—C8—O3	−0.5 (3)	C18—C17—C20—O5	−28.3 (5)
Mn1—O4—C8—C9	178.7 (3)	C18—C17—C20—N2	152.4 (3)
Mn1—N1—C15—C16	176.7 (3)	C18—C19—N1—Mn1	−174.9 (3)
C19—N1—C15—C16	1.1 (5)	C18—C19—N1—C15	0.9 (5)
C21—N3—C5—C4	−43.9 (4)	N1—C19—C18—C17	−1.6 (5)

### Hydrogen-bond geometry (Å, °)

**Table:**

*D*—H···*A*	*D*—H	H···*A*	*D*···*A*	*D*—H···*A*
N2—H2A···O4^i^	0.86	2.18	3.025 (4)	167
N2—H2B···O3^ii^	0.86	2.00	2.821 (4)	159
O6—H61···O2	0.92 (4)	1.78 (4)	2.672 (4)	162 (4)
O6—H62···O5^iii^	0.89 (4)	1.85 (4)	2.739 (4)	175 (4)
O7—H71···N3^iv^	0.91 (2)	1.96 (2)	2.838 (2)	161 (2)
O7—H72···O2^v^	0.88 (2)	1.80 (2)	2.671 (2)	170 (2)
C23—H23B···Cg2^vi^	0.96	2.90	3.649 (4)	136
C24—H24A···Cg3^vii^	0.96	2.74	3.594 (3)	149

Symmetry codes: (i) *x*−1, *y*, *z*+1; (ii) *x*, *y*, *z*+1; (iii) *x*+1, *y*, *z*−1; (iv) *x*−1, *y*, *z*−1; (v) *x*−1, *y*, *z*; (vi) −*x*, −*y*, −*z*; (vii) −*x*, −*y*, −*z*+1.

### Table 2 Comparison of the carboxylate bonds (Å) in the title compound with the corresponding values in related compounds

**Table:**

Compound	C1—O1	C1—O2	C8—O3	C8—O4
I*^a^*	1.261 (4)	1.267 (4)	1.277 (4)	1.270 (4)
II*^b^*	1.244 (4)	1.270 (4)		
III*^c^*	1.284 (2)	1.248 (2)		
	1.278 (2)	1.241 (2)		
IV*^d^*	1.267 (3)	1.258 (3)		
V*^e^*	1.263 (2)	1.240 (2)		
VI*^f^*	1.2611 (17)	1.2396 (19)		
VII*^g^*	1.2616 (17)	1.2435 (18)		
VIII*^h^*	1.2746 (18)	1.2675 (17)		
IX*^i^*	1.2682 (17)	1.2628 (17)	1.2743 (18)	1.2716 (18)
X*^j^*	1.265 (3)	1.265 (3)	1.278 (3)	1.271 (3)

Notes: (*a*) this work; (*b*) [Co(NA)_2_(H_2_O)_4_](C_7_H_4_FO_2_)_2_ (Özbek *et al.*, 2009); (*c*) [Zn(NA)_2_(C_8_H_8_NO_2_)_2_] (Tercan *et al.*,  2009); (*d*) [Ni(NA)_2_(C_7_H_4_ClO_2_)_2_(H_2_O)_2_] (Hökelek *et al.*, 2009*a*); (*e*) [Zn(DENA)_2_(C_7_H_4_BrO_2_)_2_(H_2_O)_2_] (Hökelek *et al.*,  2009*b*); (*f*) [Mn(DENA)_2_(C_7_H_4_BrO_2_)_2_(H_2_O)_2_] (Hökelek *et al.*,  2009*c*); (*g*) [Ni(DENA)_2_(C_7_H_4_ClO_2_)_2_(H_2_O)_2_] (Hökelek *et al.*, 2009*d*); (*h*) [Ni(NA)_2_(C_8_H_8_NO_2_)_2_(H_2_O)_2_] (Hökelek *et al.*, 2009*e*); (*i*) [Co(NA)_2_(C_9_H_10_NO_2_)_2_(H_2_O)_2_] (Hökelek *et al.*, 2009*f*); (*j*) [Zn(NA)_2_(C_9_H_10_NO_2_)_2_(H_2_O)_2_] (Hökelek *et al.*, 2009*g*).
